# CRISPR/Cas9-mediated deletion of a GA-repeat in human GPM6B leads to disruption of neural cell differentiation from NT2 cells

**DOI:** 10.1038/s41598-024-52675-3

**Published:** 2024-01-25

**Authors:** Hadi Bayat, Maryam Mirahmadi, Zohreh Azarshin, Hamid Ohadi, Ahmad Delbari, Mina Ohadi

**Affiliations:** 1 Iranian Research Center on Aging, University of Social Welfare and Rehabilitation Sciences, Tehran, Postal Code: 1985713834 Iran; 2 Department of Molecular Genetics, Faculty of Biological Sciences, Tarbiat Modares University, Postal Box: 331-14115, Tehran, Iran; 3 Department of Medical Genetics, Faculty of Medical Sciences, Tarbiat Modares University, Postal Box: 331-14115, Tehran, Iran; 4 Department of Exomine, PardisGene Company, Tehran, Postal Code: 1917635816 Iran; 5 School of Physics and Astronomy, University of St Andrews, St Andrews, KY16 9SS UK

**Keywords:** Gene expression, Gene regulation, Evolution, Genetics, Molecular biology

## Abstract

The human neuron-specific gene, GPM6B (Glycoprotein membrane 6B), is considered a key gene in neural cell functionality. This gene contains an exceptionally long and strictly monomorphic short tandem repeat (STR) of 9-repeats, (GA)9. STRs in regulatory regions, may impact on the expression of nearby genes. We used CRISPR-based tool to delete this GA-repeat in NT2 cells, and analyzed the consequence of this deletion on GPM6B expression. Subsequently, the edited cells were induced to differentiate into neural cells, using retinoic acid (RA) treatment. Deletion of the GA-repeat significantly decreased the expression of GPM6B at the RNA (*p* < 0.05) and protein (40%) levels. Compared to the control cells, the edited cells showed dramatic decrease of the astrocyte and neural cell markers, including GFAP (0.77-fold), TUBB3 (0.57-fold), and MAP2 (0.2-fold). Subsequent sorting of the edited cells showed an increased number of NES (*p* < 0.01), but a decreased number of GFAP (*p* < 0.001), TUBB3 (*p* < 0.05), and MAP2 (*p* < 0.01), compared to the control cells. In conclusion, CRISPR/Cas9-mediated deletion of a GA-repeat in human GPM6B, led to decreased expression of this gene, which in turn, disrupted differentiation of NT2 cells into neural cells.

## Introduction

GPM6B (glycoprotein membrane 6B) is a membrane protein, belonging to the proteolipid protein (PLP) family^1^ and almost exclusively expressed in the brain, with high levels of expression in astrocytes, oligodendrocytes, and neurons^2,3^. GPM6B has a pivotal role in axon growth and guidance^4^, stress response^5^, and cell–cell communication^6^. Deletion of this gene in male mice, using CRISPR/Cas9 technology, may correlate with psychiatric disorders^1^. The expression of GPM6B is decreased in human glioblastoma, which may have consequences for dedifferentiation and tumor progression^7^.

Short tandem repeats (STRs) constitute 5% of the human genome^8^. They are a significant source of variation across species, as reflected in the highly polymorphic nature and plasticity of these extensively abundant genetic elements. Among numerous biological functions (for a review, see^9^), STRs modulate gene expression^10,11^ and translation^12^, and it is likely that a number of STRs function as evolutionary switch codes for speciation^13,14^. Comparative analyses support adaptive evolutionary patterns for a number of STRs, and the co-occurrence of divergent alleles at these loci with major human disorders^15–19^. Moreover, STR length influences the expression of quantitative trait loci (eQTL) associations^20,21^. Recently, it has been elucidated that STRs may significantly impact on transcription factor (TF) binding affinity at target sites^22^. According to high-throughput protein-binding assays, it is estimated that approximately 90% of TFs can bind to, at least one kind of STR in eukaryotes^23^. Due to their highly mutable nature, STRs are considered dynamic *cis*-regulatory elements that can function as rheostats to increase the local binding affinity of TFs^22^.

A subset of human STRs reach lengths of ≥ 6 repeats in the critical core promoter and 5′ untranslated region (UTR), i.e., the interval between − 100 and + 100 of the transcription start site, which may be of importance concerning adaptive evolution^24,25^. One of the longest GA-repeats in this interval resides in the regulatory region of GPM6B. In our previous work in a sample of 600 humans, encompassing various neurological disorders and controls, the GPM6B GA-repeat was strictly monomorphic, of 9-repeats^26^.

Evidence of the evolutionary and biological impact of STRs is largely circumstantial and limited to approaches such as comparative genomics, association studies, genotype–phenotype analyses, in vitro gene reporter studies, and bioinformatics studies.

CRISPR/Cas9, as a versatile gene editing technology, has provided the capacity to precisely target specific regions in the human genome^27^. Limited instances of CRISPR/Cas9-mediated targeting of large STR expansions have been reported in a number of neurological and movement disorders^28,29^.

In the current study, we used high-specificity CRISPR/Cas9 tool^27,30^ to precisely delete the GPM6B GA-repeat in NT2 cells. The consequence of this deletion on the expression of *GPM6B* was evaluated at the RNA and protein levels, using qRT-PCR and western blotting assays, respectively. Furthermore, differentiation of edited NT2 cells into neurons was evaluated under retinoic acid (RA) treatment, using several markers: as follows: NESTIN (NES) (a pluripotency marker)^31^, Glial fibrillary acidic protein (GFAP) (a specific astrocytic marker)^32^, and neuronal-specific tubulin isoform 3 (TUBB3) and microtubule-associated protein 2 (MAP2) (as pan-neuronal markers)^33^.

Here, we provide prime evidence of the role of GPM6B in the differentiation of human neural cells, mediated by the GA-repeat. These data are also direct evidence of the impact of an STR at the biological level, achieved by CRISPR/Cas9.

## Results

### In-silico study: positive correlation of GPM6B with neural cell differentiation

**Figure 1:**
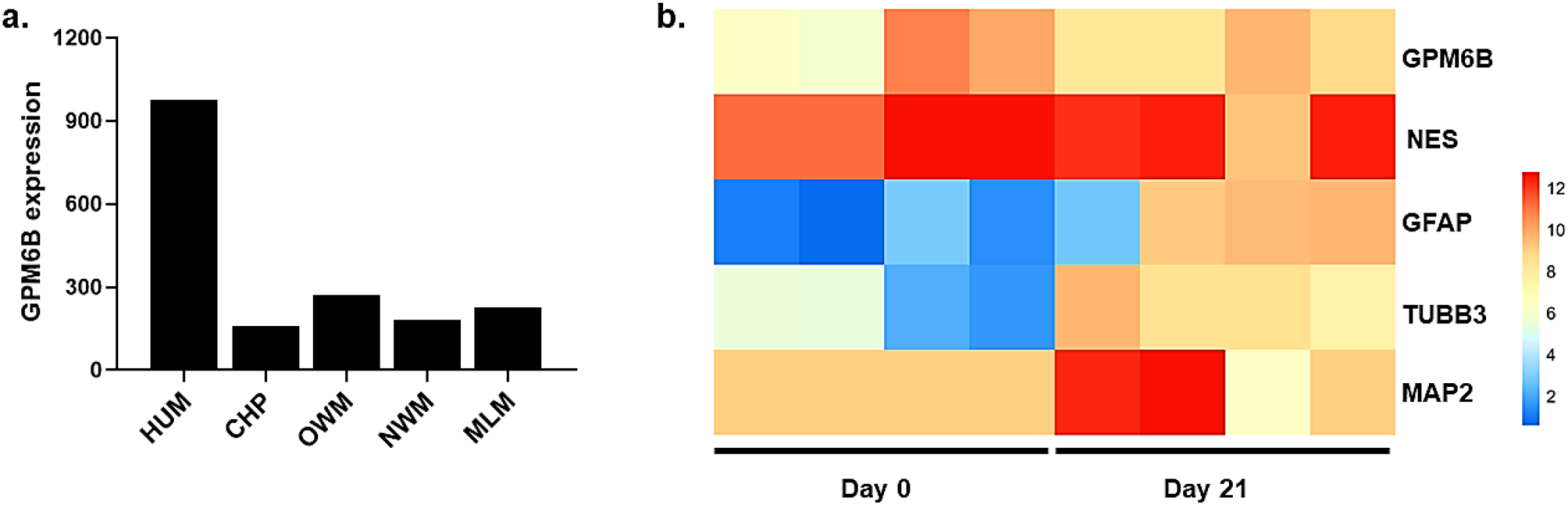
In-silico analysis of GPM6B expression. (**a**) Across several primates, the expression of GPM6B was at its highest level in the human brain. (**b**) Our analysis of the RNA-seq datasets retrieved from GEO showed a positive correlation between GPM6B and neural cell differentiation markers, such as GFAP, TUBB3, and MAP2, after differentiation of NT2 cells into neural cells (21 days under RA treatment). HUM, human; CHP, chimpanzee; OWM, Old-World monkeys; NWM, New-World monkeys; MLM, mouse lemur.

GPM6B showed the highest level of expression in the human brain, compared to other primates in the AceView database^34^ (Fig. 1a). The human GPM6B expression ratio in comparison to chimpanzee, Old-World monkeys, New-World monkeys, and lemurs was 6.1-, 3.6-, 5.4-, and 4.3-fold, respectively.

NT2 cells under RA treatment can differentiate into neural cells. To elucidate the expression status of GPM6B in neural cell differentiation, we performed an in-silico analysis of RNA sequencing (RNA-seq) datasets retrieved from the Gene Expression Omnibus (GEO) database (Table S1). The expression pattern of GPM6B showed a positive correlation with GFAP, TUBB3, and MAP2 (Fig. 1b). Based on these observations, we hypothesized that GPM6B is important for the differentiation of neural cells from NT2 cells.

### CRISPR/Cas9-mediated deletion of the GA-repeat in NT2 cells

**Figure 2:**
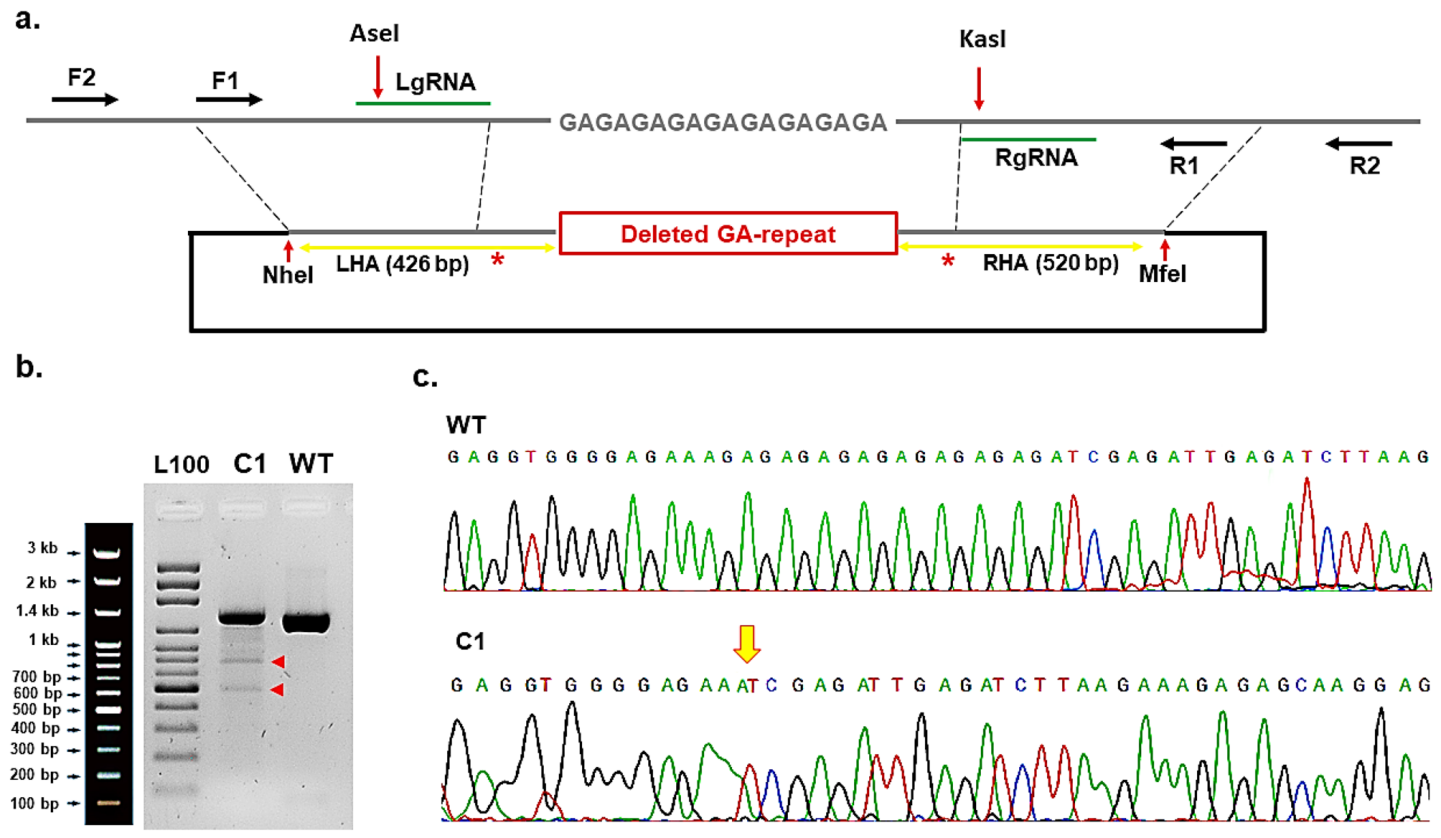
Targeting and deleting the GA-repeat at the GPM6B gene using eSpCas9(1.1). (**a**) Schematic overview of the designed strategy to target and delete the GA-repeat. Green lines: gRNA locations; black arrows: primer location; red arrows: restriction enzymes sites; red stars: introduced mutations at PAM sites; yellow double arrows: LHA (426 bp) and RHA (520 bp); and red rectangle: the deleted GA-repeat. (**b**) T7E1 assay confirmed the efficiency of the designed gRNAs, targeting the GA-repeat at GPM6B gene. The original gel image is presented in Supplementary Fig. Xa. (**c**) Sanger sequencing of the wild-type GPM6B and the edited C1 confirmed successful deletion of the GA-repeat. Yellow arrow, deletion site of GA-repeat; F, forward primer; R, reverse primer; LgRNA, left gRNA; RgRNA, right gRNA; LHA, left homology arm; RHA, right homology arm; L100, ladder 100 bp; C1, edited clone 1; WT, wild-type.

To evaluate the potential effect of the GA-repeat on GPM6B expression, we first designed a protocol using the CRISPR/Cas9 editing tool to delete the GA-repeat, by applying homology-direct repair (HDR) pathway (Fig. 2a). The donor vector, peGPM6B, comprised a template without the GA-repeat at the target site. We mutated protospacer adjacent motif(PAM) sites in the donor vector to prevent re-targeting of the target site or unwanted targeting of peGPM6B. PAM mutations were introduced in both the donor vector and scrambled donor vector. We designed guide RNAs(gRNAs) at specific sites, but did not disrupt TF binding sites in the regulatory region of the GPM6B gene (Fig. 2a). Moreover, we used eSpCas9(1.1) to express high-specificity spCas9, which significantly decreased off-target effects. To evaluate the efficiency of the designed gRNAs at the target site, the results of the T7E1 assay indicated that the editing efficiency in the pool cells was 15.5% (Fig. 2b). The edited pool cells were serially diluted (1 cell/100 µL) and cultured in a 96-well plate to obtain a homogenous single clone. Subsequently, we used specific primer sets to determine the successfully edited cells. First, to exclude the clones in which the donor vector was randomly integrated, we used the GPM6B F2 and R2 primer set outside the homology arms (Fig. 2a). Next, nested ARMS-PCR was applied to obtain PCR products, using specific primer sets (GPM6B-IF and GPM6B-R1; GPM6B-mutIF and GPM6B-R1). Clone (C) 1 and C4 were successfully amplified, using the F2 and R2 primer set (Fig. S1a). Nested ARMS-PCR showed that both clones were successfully edited (Fig. S1b). Finally, to confirm the accuracy of the donor template KI, Sanger sequencing was performed, and successful deletion of the GA-repeat at the target site was confirmed (Fig. 2c). We used the same PCR-based strategy to detect successful KI in scrambled clones (SCs). Among all tested clones, SC3 showed positive results and was selected for downstream experiments (Data not shown). To confirm the accuracy of SC3, mutations at PAM sites were confirmed, using Sanger sequencing (Fig. S1c).

### Deletion of the GA-repeat significantly reduced the expression of the GPM6B gene

**Figure 3:**
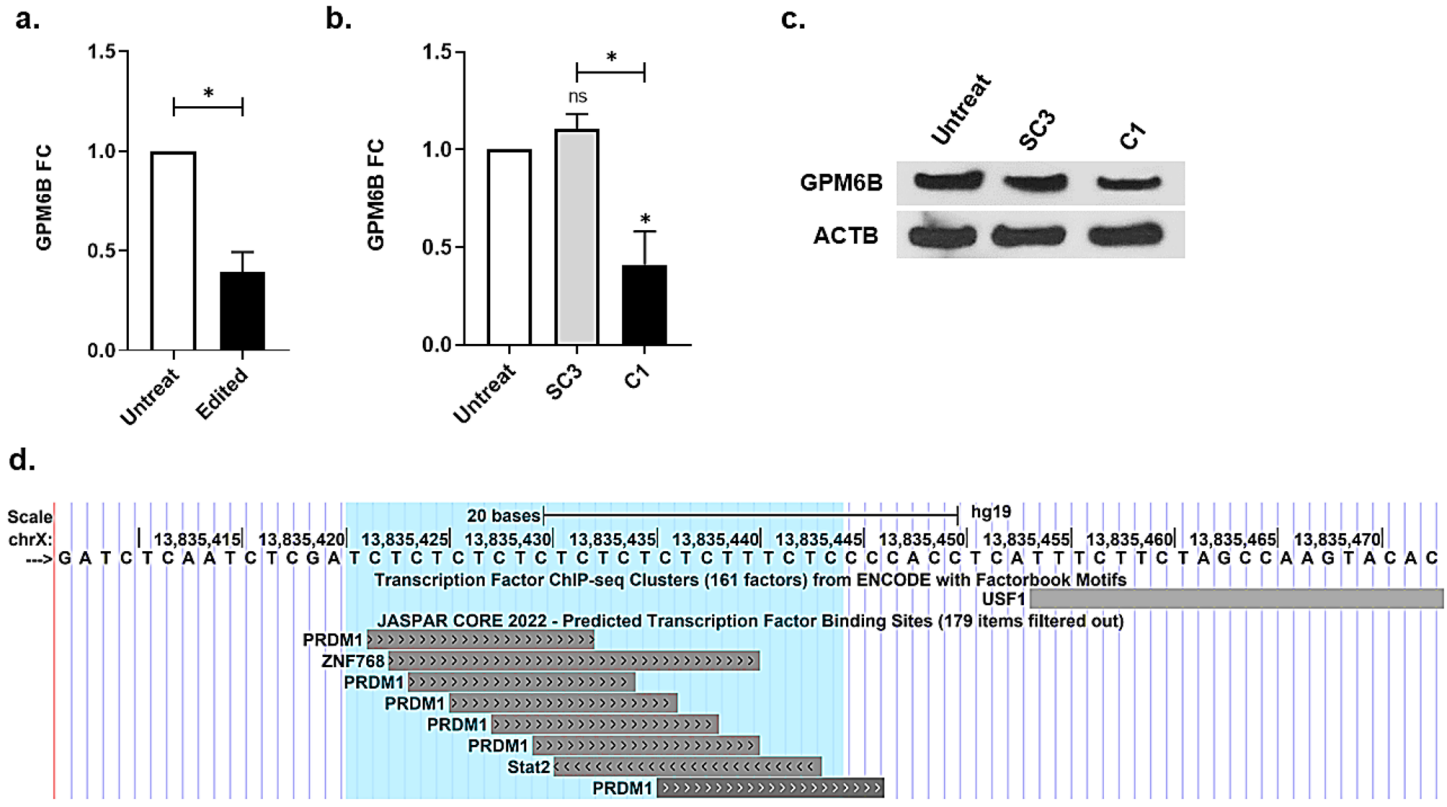
Measuring the expression level of the edited GPM6B gene at the RNA and protein levels in NT2 cells. (**a**) The expression level of GPM6B was evaluated in the untreated and edited NT2 pool cells, using qRT-PCR. The expression level of GPM6B decreased significantly in the edited pool cells (*p* < 0.05). (**b**) The expression of GPM6B was assessed in the isolated single clones, using qRT-PCR. The expression of GPM6B was significantly decreased in the C1 cells, compared to the untreated and the SC3 cells. (**c**) Western blotting assay confirmed that the expression level of GPM6B was decreased in the C1 cells more efficiently, compared to the untreated and SC3 cells. The original blot images are presented in Supplementary Fig. Xd. (**d**) the predicted TF binding sites at GA-repeat site and its flanking sequence, is presented based on JASPAR CORE 2022 and ChIP-seq data from ENCODE. The GA-repeat site is highlighted with light blue.

We used the CRISPR/Cas9 gene editing tool to precisely delete (GA)9 and evaluated its effect on GPM6B expression. For this purpose, GPM6B was targeted in NT2 cells, which can be further differentiated into neural cells by RA treatment. The expression level of GPM6B was measured in the untreated and edited pool cells by qRT-PCR, and the results showed a significant reduction in GPM6B expression in the edited cells (Fig. 3a). Furthermore, we assessed the expression level of GPM6B in the single clones, using qRT-PCR. The expression of GPM6B was not statistically different between the untreated and SC3 cells. However, the expression level of GPM6B in the C1 cells significantly lower than that in untreated and SC3 cells (*p* < 0.05) (Fig. 3b). The protein expression of GPM6B was measured to confirm the accuracy of the qRT-PCR results. Our results showed that the expression of GPM6B in the SC3 and C1 cells decreased by 14.8% and 49%, respectively, compared to that in untreated cells. Moreover, the expression of GPM6B in C1 decreased by 34.1% compared to SC3 (Fig. 3c). Our observation of data retrieved from the ENCODE project in the UCSC database revealed that the GPM6B (GA)9 is a potential binding site for PRDM1, ZNF768, and Stat2 TFs (Fig. 3d). Moreover, this dinucleotide STR is close to the USF1 TF binding site, where the binding affinity was confirmed using chromatin immunoprecipitation sequencing (ChIP-seq) (Fig. 3d).

### Deletion of the GA-repeat in GPM6B disrupted optimal differentiation of NT2 cells into neural cells

**Figure 4:**
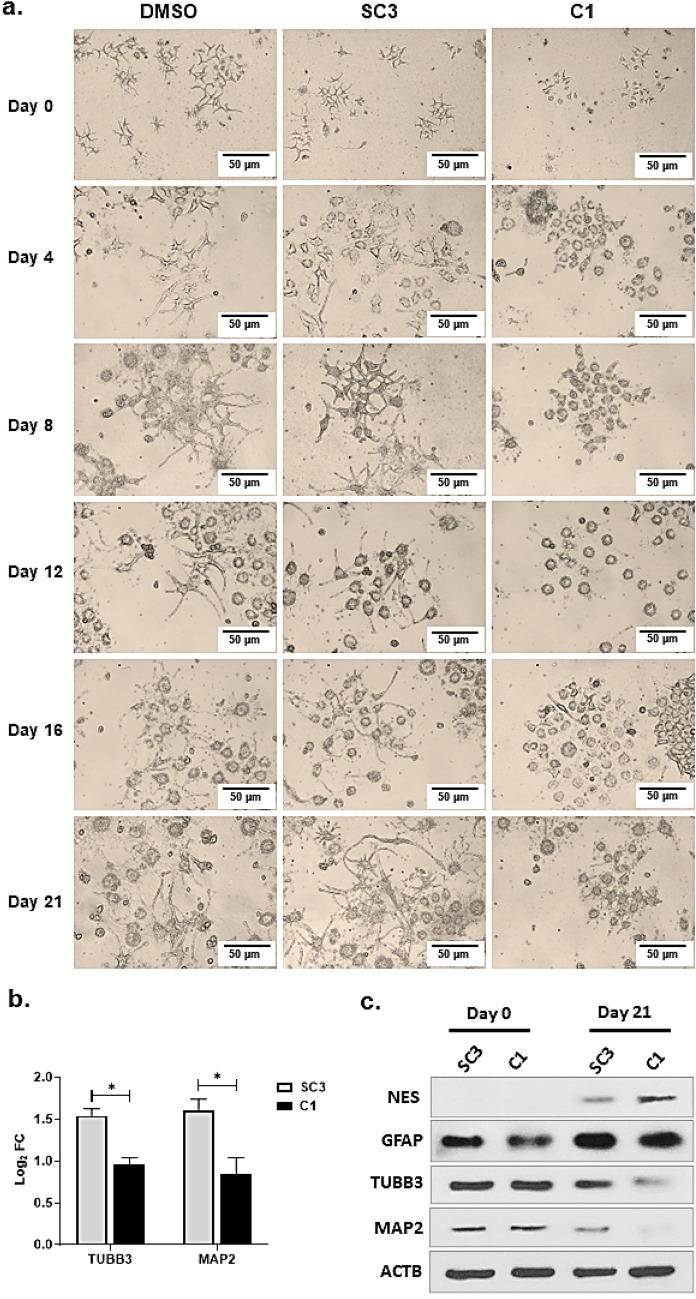
Decreased level of the GPM6B due to the GA-repeat deletion reduced differentiation of NT2 cells. (**a**) Differentiation of NT2, SC3, and C1 cells was monitored under RA treatment on Days 0, 4, 8, 12, 16, and 21. DMSO was used to dissolve RA, and was added to NT2 cells to evaluate its background effect of cell differentiation. On different days, the morphological changes and cell communication among differentiated cells were observed in NT2 and SC3 cells, whereas the morphology of C1 cells mainly remained undifferentiated. (**b**) The expression of TUBB3 and MAP2 was measured at the RNA level, using qRT-PCR. The expression of these genes was significantly decreased in the C1 compared to the SC3 cells. (**c**) The expression of neural markers was measured on Day 0 and Day 21 of RA-treatment, using western blotting (the original blot images are presented in Supplementary Fig. Xe). The expression of NES as an indicator of neural progenitor cells increased, while the expression of GFAP, TUBB3, and MAP2, as differentiated neural markers, were decreased in the C1 compared to the SC3 cells.

Following RA treatment, NT2 cells differentiate into neural cells. We monitored the DMSO-treated, SC3, and C1 NT2 cells on Days 0, 4, 8, 12, 16, and 21 after RA treatment. The NT2 cells treated with DMSO treatment and the SC3 cells under RA treatment showed neural morphology on different days, whereas the differentiation of the C1 cells under RA treatment was disrupted to a considerable extent (Fig. 4a). Our results showed that DMSO and the introduced mutations at the PAM sites had no significant effect on the NT2 cell differentiation. At the same time, deletion of the (GA)9 decreased the expression of the GPM6B and consequently mainly disrupted the differentiation of the modified NT2 cells.

To study the link between the reduced amount of GPM6B and the deficient differentiation of the NT2 cells into neural cells, the expression of TUBB3 and MAP2 was measured, using qRT-PCR. The expression of both markers decreased significantly in the C1 cells, compared to that in the SC3 cells (*p* < 0.05) (Fig. 4b). Moreover, the protein expression of NES was assessed before and after RA treatment on Day 0 and Day 21. The results showed that the C1 cells had higher expression levels (approximately 2.35-fold) than the SC3 cells (Fig. 4c). To that end, we first measured the ratio of Day 21/Day 0 for the SC3 and C1, and then calculated the ratio of the C1 to the SC3. Accordingly, the expression of NES was increased 2.3-fold in the C1 cells compared to that in the SC3 cells. This observation confirmed that in the C1 cells, the number of undifferentiated cells was higher than that of the SC3 cells. To study whether the effect of decreased expression of GPM6B on the differentiation of NT2 cells to neural cells (under RA treatment) is due to deletion of (GA)9, the expression levels of GFAP, TUBB3, and MAP2 were evaluated at the protein level. The results indicated that GFAP, TUBB3, and MAP2 expression decreased by 0.77-, 0.57-, and 0.2-fold, respectively, compared to the SC3 (Fig. 4b). Based on these findings, the reduced expression level of GPM6B, due to the specific deletion of (GA)9, consequently decreased the differentiation of NT2 cells under RA treatment.

**Figure 5:**
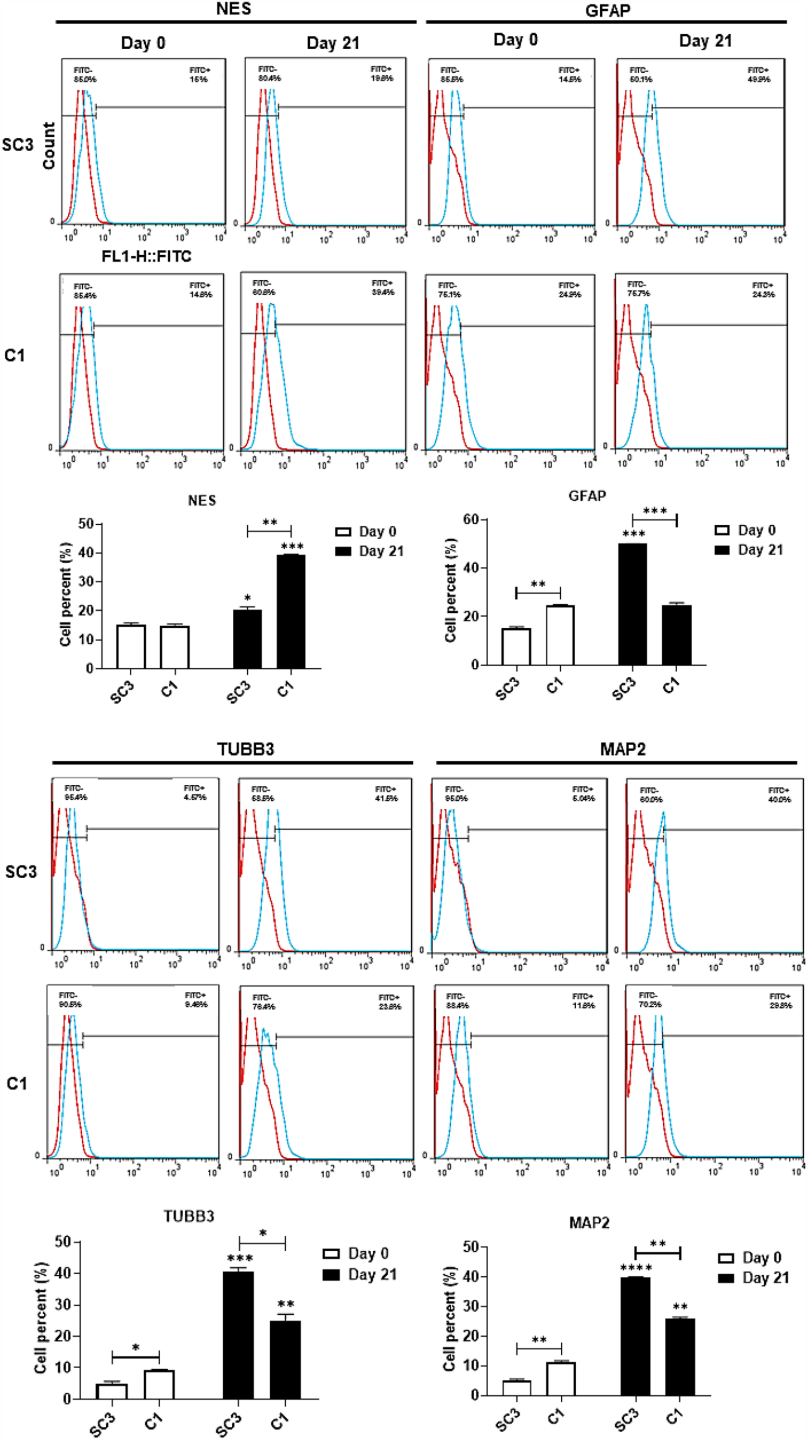
Disrupted differentiation of NT2 cells to astrocytic and neural cells as a result of GA-repeat deletion and GPM6B downregulation. Deletion of the GA-repeat in the regulatory region of GPM6B decreased the expression of this gene. Consequently, the number of differentiated cells expressing GFAP, TUBB3, and MAP2 decreased significantly in the C1 compared to the SC3 cells.

To count differentiated cells in each experiment under RA treatment, specific cell markers, such as NES, GFAP, TUBB3, and MAP2, were labeled, using specific antibodies and sorted by flow cytometry on Day 0 and Day 21. The number of SC3 and C1 cells expressing NES significantly increased compared to Day 0 (*p* < 0.05 and *p* < 0.001, respectively). Moreover, the number of cells expressing NES increased substantially in the C1 cells compared to that in the SC3 cells on Day 21 (*p* < 0.01). The number of undifferentiated cells in the C1 was higher than that in the SC3, confirming that a decreased level of GPM6B reduced the differentiation of NT2 cells to neural cells. The number of cells expressing GFAP on Day 21 significantly increased in the SC3 cells (*p* < 0.001) but not at C1, compared to their counterparts on Day 0. Furthermore, the number of the SC3 cells expressing GFAP was significantly higher than that in the C1 cells on Day 21 (*p* < 0.001). It can be concluded that decreased levels of GPM6B disrupted differentiation of the NT2 cells into neural cells expressing GFAP. Our findings indicated that the number of cells expressing TUBB3 on Day 21 was significantly increased compared to the cells on Day 0 in the SC3 (*p* < 0.001) and the C1 (*p* < 0.01). In addition, the number of differentiated cells expressing TUBB3 was significantly lower in the C1 cells than in the SC3 cells (*p* < 0.05). Similar to the TUBB3 findings, the number of cells expressing MAP2 was significantly increased in the SC3 and the C1 cells on Day 21, compared to the cells on Day 0 (*p* < 0.0001 and *p* < 0.01, respectively). Moreover, the number of differentiated cells expressing MAP2 decreased significantly in the C1 compared to the SC3 cells (*p* < 0.01) (Fig. 5). Taken together, our findings elucidated that the reduced expression level of GPM6B due to the deletion of the GA-repeat significantly reduced NT2 cell differentiation to neural cells.

## Discussion

In this study, we employed high-specificity CRISPR/Cas9 to precisely delete a GA-repeat in the regulatory region of the human neuron-specific gene,GPM6B, aimed at studying the consequences of this deletion. We detected significant decrease in the expression level of GPM6B, leading to disruption of NT2 cells differentiating into neural cells, as a result of the GA-repeat deletion.

Studies in mice have shown that GPM6B is expressed in both neurons and glial cells^35^, and is pivotal for the regulation of various functions in the brain, such as controlling synaptic function by downregulation of the serotonin transporter^36^. In human, decreased levels of GPM6B are linked to poor decision-making and enhanced suicidal behaviors^37^. Moreover, the expression of GPM6B was decreased in higher-grade glioma cells, and patients with lower expression levels of this gene showed an increased risk of glioma development^7^.

Previously, Drabeck et al., reported that GPM6B was upregulated during osteoblast differentiation. Lentiviral-mediated knockdown of this gene, using shRNAs, showed depletion of cytoskeleton organization and biogenesis in human mesenchymal stem cells through downregulation of PDLIM7, a key regulator of bone morphogenesis^6^. Moreover, the potential effect of GPM6B was reported to promote the differentiation of smooth muscle cells through the activation of TGF-β-Smad2/3 signaling. Activation of this signaling pathway, in turn, facilitated the expression of GPM6B and increased differentiation of smooth muscle cells^2^. To our knowledge, our study is the first to report the importance of GPM6B in the differentiation of human neural cells, achieved by differentiating NT2 stem-like cells into neural cells.

Repetitive elements have shown a high prevalence in non-coding regions, especially in *cis*-regulatory elements that mainly regulate gene expression and result in phenotypic evolution^38^. By using electrophoretic mobility shift assay, Horton et al.^22^, reported that STRs have potential to change the binding affinity of TFs at the target sites. In this line, variants of the polymorphic GA-repeats in the core promoter of the embryogenesis genes, alter the expression level of reporter genes in cell models^11^.

However, the bulk of evidence on the impact of STRs on various biological and evolutionary processes is largely circumstantial. Approaches such as genome editing protocols at these loci have been exclusively reported in conjunction with the treatment of large trinucleotide repeat expansions in neurological disorders^39–41^. To date, these protocols have not been used to examine the impact of STRs on the biological level and biological length ranges. To our knowledge, our approach in using high-specificity CRISPR/Cas9 to precisely delete the GPM6B GA-repeat in the NT2 cells, is the first study to directly confirm the biological effect of a STR. Our findings indicate that the differentiation of GPM6B-edited cells was disrupted, and cell morphology differed from that observed in the control cells.

In view of the strict monomorphism of the human GPM6B GA-repeat, at 9-repeats, enrichment of exceedingly rare alleles of this repeat in the disease compartment, and the functionality of the repeat in neural cell differentiation, it is envisioned that the (GA)9 is the optimal repeat length for human evolution.

The GPM6B GA-repeat is a potential target for binding TFs, such as PRDM1, ZNF768, and STAT2. ZNF768 has RNA-binding activity, and is probably involved in gene transcription by RNA polymerase II^42^. PRDM3 is a member of the PRDM protein family, and a mouse model lacking this protein showed a deficiency in neuronal differentiation^43^. USF1 is a basic helix-loop-helix leucine zipper family member that activates transcription through E-box motifs^44^. PRDM1 and STAT2 are involved in immune responses controlling viral infections^45,46^. These data further support our findings that deletion of the GA-repeat significantly decreased the expression of GPM6B at the RNA and protein levels. Based on our findings, deletion of the GPM6B GA-repeat may affect the potential binding affinity of the proposed TFs, and consequently decrease the expression of GPM6B. Furthermore, the interaction of GPM6B with differentiation factors and signaling pathways, such as TGF-β, may be a key factor in the differentiation of various cell lineages, such as neural cells. Future studies are warranted to understand the mechanisms underlying human neural cell differentiation through the GPM6B and its GA-repeat.

## Conclusion

Using high-specificity CRISPR/Cas9, we provide evidence of the role of GPM6B and a GA-repeat in its regulatory region, in human neural cell differentiation. While the bulk of evidence on the impact of STRs is circumstantial, our approach provides prime direct evidence of such an impact at the biological level.

## Materials and methods

### Materials

All the primers and oligonucleotides were ordered from GeneScript (Jiangsu, China). DNA Polymerase High Fidelity (KBC Hi-Fid™ DNA Polymerase) Enzyme was purchased from Kowsar Biotech (Cat. #KBC-520103-100; Tehran, Iran). Lipofectamine™ 2000 Transfection Reagent (Cat. #11668019) and the restriction enzymes NheI (Cat. #ER0972), MfeI (Cat. #ER0751), AseI (Cat. #ER0751), KasI (Cat. #ER0751), and BbsI (Cat. #ER1011) were obtained from Thermo Scientific (Massachusetts; United States). The cell culture materials: high glucose DMEM (Cat. #11965092), penicillin-streptomycin (Cat. #15140122), fetal bovine serum (FBS) (Cat. #11550356), and trypsin-EDTA (Cat. #25200056) were purchased from GIBCO (Massachusetts; USA). Phosphate Buffered Saline (PBS) was obtained from Thermo Scientific (Cat. #10010002; Massachusetts; USA). Non-essential amino acids were ordered from Cyagen Biosciences (Cat. #NEAA-10201-50; Jiangsu; China). Trans-retinoic acid (RA) (Cat. #554720), DMSO (Cat. #D2650), Bovine serum albumin (BSA) (Cat. #B8667), Paraformaldehyde (Cat. #158127) and antibiotics, including Hygromycin B (Cat. #10843555001) and Puromycin (Cat. #P8833) were ordered from Sigma-Aldrich (Missouri; USA). The experimental kits, such as the T7 endonuclease 1 (T7E1) kit (Cat. #M0302S), genomic DNA Extraction Blood DNA Kit (Cat. #FABGK 100), and DNase I, RNase-free kit (Cat. #FEREN0521), were purchased from New England Biolabs (Ipswich; USA), Favorgen (Pingtung; Taiwan), and Thermo Scientific (Massachusetts; USA), respectively. RIPA buffer was obtained from Cell Signaling Technology (Cat. #9806; Massachusetts; USA). Polyvinylidene difluoride membrane (PVDF) (Cat. #PI88518) and Pierce ECL Western Blotting Substrate (Cat. #PI32106) were purchased from Thermo Scientific (Massachusetts; USA).

### In-silico analysis

RNA-seq datasets of NT2 cell lines and differentiated NT2 cells with RA were retrieved from the GEO database. After checking quality control, the reading adaptor was eliminated by cutadapt (RRID:SCR_011841)^47^. Subsequently, HISAT2 (RRID:SCR_015530)^48^ was used to map the RNA-seq reads in the reference genome, and featureCounts (RRID:SCR_012919)^49^ was employed to count the number of reads per gene. Removal batch effect and normalization were done using LIMMA (RRID:SCR_010943) and sva package (RRID:SCR_012836)^50,51^. Finally, Heatmap plots were drawn using ggplot2 (RRID:SCR_014601)^52^.

### Plasmid construction

All the primers and oligonucleotides used in this study are listed in Table S2. Polymerase High Fidelity (HiFi) Enzyme was used for all polymerase chain reaction (PCR) experiments. The amplified products were cloned into pGEM®-T Easy Vector Systems (Promega; USA). Finally, the accuracy of amplified products was validated, using the Sanger Sequencing 3500 Dx Genetic Analyzer (Applied Biosystems; USA).

To generate the GPM6B Donor vector, the sequence of the target site in the GPM6B gene was amplified, using a GPM6B-cloning primer set. The amplified product was sub-cloned into pGL4.14[luc2 Hygro] (Cat. # E6691; Promega; USA) between NheI and MfeI restriction sites (called p4GPM6B). The sequence of target sites without the (GA)9-STR and containing a mutation at PAM sites were synthesized and subcloned into p4GPM6B between AseI and KasI restriction sites to generate a donor vector containing (GA)9-less template (Called peGPM6B). To evaluate the effects of mutations at PAM sites, we synthesized scrambled donor vector sequence containing (GA)9 and harboring mutations at PAM sites. The synthesized scrambled sequence was subcloned into p4GPM6B between AseI and KasI restriction sites (peGPM6SB).

The gRNAs were designed using a user-friendly CRISPOR (RRID:SCR_015935) online tool^53^. The sequence of synthesized gRNAs is listed in Table S2. In this study, we used all-in-one eSpCas9(1.1) (RRID:Addgene_71814; USA) to express high specificity SpCas9 and designed gRNAs^54,55^. As previously reported, the synthesized oligonucleotides re-annealed and cloned into eSpCas9(1.1) at the BbsI restriction site^56^. The accuracy of the cloned gRNAs was confirmed by Sanger sequencing.

### Cell culture and differentiation

NTERA-2 (NT2) cell line (Cat. #ACC-527, RRID:CVCL_0034; was purchased from DSMZ-German Collection of Microorganisms and Cell Cultures GmbH), resembling characteristics of human neuronal progenitor cell, cultured in high glucose DMEM supplemented with 1% penicillin-streptomycin, and 10% FBS under optimal growth condition (at 37 °C, 5% CO_2_, 80% humidity). The cultured cells were passaged every three days at a density of 8 $\times$ 10^3^ cells/cm^2^.

NT2 cells can be differentiated into neuronal cells (NT2N) under RA treatment^57^. To do this, we applied the previously introduced protocol^58^. In brief, we generated a 25 mg/mL RA solution diluted in DMOS. Subsequently, 1.5 × 10^5^ NT2 cells were cultured in a 10 mL complete medium in a T75 cell culture flask under optimal growth conditions. The seeded cells were treated with RA twice weekly (1 $\times$ 10^–2^ RA/mL), and the medium was changed each week. The differentiation process was monitored every 3 days. Total RNA was extracted from differentiated cells on days 0, 8, and 21. Moreover, Total protein was extracted on Day 0 and 21 of differentiation. To validate the differentiation process, the expression of two major neural cell markers, TUBB3 and MAP2, was measured, using the qRT-PCR (Data not shown).

### Gene editing and single-cell cloning

5 $\times$ 10^4^ NT2 cells were seeded in a 24-well plate. After 24 h, an equal amount of both constructs expressing LgRNA and RgRNA were transfected into NT2 cells, using Lipofectamine™ 2000 Transfection Reagent according to the manufacturer’s manual. The transfectants were incubated at optimal growth conditions up to 72 h. The editing efficiency was evaluated, using the T7E1 assay according to the manufacturer’s protocol^59^.

To obtain a single edited cell clone, we seeded 5 $\times$ 10^4^ NT2 cells in a 24-well plate. An equal amount of LgRNA, RgRNA, and peGPM6B/peGPM6SB were transfected into seeded cells, using Lipofectamine™ 2000 Transfection Reagent. 24 h after transfection, the transfectants were treated with both Hygromycin B (200 µg/mL) and Puromycin (0.3 µg/mL) for 48 h to ensure obtaining the cells containing our transfected plasmids. Subsequently, the remaining cells were detached using Trypsin-EDTA 0.25%. The edited cells were diluted in 1 cell/100 µL enriched medium, including high glucose DMEM, 20% FBS, 1% non-essential amino acids, and 1% penicillin-streptomycin. The single cells were incubated under optimal growth conditions for 14 days to obtain a single clone. Then, the single clones were selected under an inverted microscope and used for downstream experiments.

### Genomic DNA purification and junction PCR

To purify genomic DNA (gDNA), a genomic DNA Extraction Blood DNA Kit was used. Junction PCR was performed, using appropriate primer sets to confirm the accuracy of knock-in (KI) of donor templates (Table S2). Specific primers (F2 and R2) were located upstream and downstream of homology arms (LHA and RHA) in gDNA and were used to identify the correctly edited single clones.

### RNA extraction, cDNA synthesis, and qRT-PCR

To quantify the expression level of target genes, total RNA was extracted from treated NT2 cells. The extracted RNAs were treated with DNase I. The expression of all target genes was measured by specific primer sets (Table S2). HPRT was used as an internal control to normalize the target genes' expression level, and the 2^−ΔΔct^ formula was used to calculate the mRNA expression level. The qRT-PCR was performed using StepOne Software (RRID: SCR_014281).

### T7E1 assay

T7E1 was used to evaluate the efficiency of designed gRNAs. To that end, primers (F2 and R1) were used to amplify the target region using HiFi-Enzyme. To generate heteroduplexes, the products were incubated in re-annealing condition as follow: stored at 95 °C for 5 min, annealing to 85 °C with a ramp of – 2 °C/s, annealing to 25 °C with a ramp of − 0.3 °C/s, and finally stored at 4 °C. The heteroduplexes were treated with T7E1 at 37 °C for 20 min. The digested products were monitored on 2% agarose gel, and the band density was measured using GelAnalyzer 23.1 (available at www.gelanalyzer.com).

### Western blotting assay

To evaluate the expression of candidate genes, total protein from edited cells and its controls were extracted on ice, using RIPA buffer (Cell Signaling Technology; United States). Western blot was performed as previously described^60^. In brief, the concentration of extracted protein was determined at 490 nm by Bradford assay. The total proteins were separated by size on a 10% SDS-polyacrylamide gel electrophoresis. After that, the electrophoresed products were transferred to a PVDF, and a 5% BSA was used for blocking at room temperature. The membranes were incubated overnight with an optimized concentration of primary antibodies at 4 °C. After that, the treated membranes were washed with Tris-buffered saline-Tween solution and incubated with secondary HRP-conjugated antibodies. The specific protein bands were stained using Pierce ECL Western Blotting Substrate. Finally, the densitometry of the photographed western blot was measured using ImageJ (RRID:SCR_003070). The primary and secondary antibodies used for western blot are listed in Table S3.

### FACS

The undifferentiated and differentiated cells were separately detached, using Trypsin–EDTA 0.25% for 3 min at 37 °C. Then, the detached cells were centrifuged at 135 $\times$ g for 5 min. The supernatant was discarded, and the cell pellets were resuspended in 500 µL paraformaldehyde 4% to fix cells for 20 min. After that, 1 mL ice-cold PBS 1× was added to mixtures and centrifuged at 135 $\times$ g for 5 min at 4 °C. The supernatant was discarded, and 1:100 diluted primary antibody in PBS was added to the cells and incubated at 37 °C for 2 h. The primary antibody was washed with 500 µL PBS, and the supernatant was discarded. Then, secondary antibodies were added and incubated for 45 min in a dark room. Finally, the mixtures were washed with 500 µL PBS, and all the samples were analyzed with BD FACSCalibur Flow Cytometry System (RRID:SCR_000401). The raw data were quantified with FlowJo v.10 (RRID: SCR_008520). The primary and secondary antibodies used in this study are listed in Table S3.

### Statistical analysis

To evaluate all obtained quantitative results, we used GraphPad Prism (RRID: SCR_002798). The significance of quantified changes was measured using a t-test. Hence, *p* < 0.05 were considered statically significant. All the experiments were performed in two replicates.

### Supplementary Information

Supplementary Information.

## Data Availability

All data generated or analyzed during this study are included in this published article [and its supplementary information files].

## Abbreviations

CRISPR/Cas9: Clustered regularly interspaced palindromic repeats /Cas-associated protein 9
eQTL: Expression quantitative trait loci
GFAP: Glial fibrillary acidic protein
GnomAD: Genome Aggregation Database
GPM6B: Glycoprotein membrane 6B
MAP2: Microtubule-associated protein 2
NCD: Neurocognitive disorder
RA: Retinoic acid
STR: Short tandem repeats
TF: Transcription factor
TOPMed: Trans-Omics for Precision Medicine
TSS: Transcription start site
TUBB3: Tubulin Beta 3 Class III
UTR: Untranslated region

## References

[1] Sanchez-Roige S (2022). A mutant allele of glycoprotein M6-B (Gpm6b) facilitates behavioral flexibility but increases delay discounting. Genes Brain Behav..

[2] Zhang X (2019). Glycoprotein M6B interacts with TbetaRI to activate TGF-beta-Smad2/3 signaling and promote smooth muscle cell differentiation. Stem Cells.

[3] Choi KM, Kim JY, Kim Y (2013). Distribution of the immunoreactivity for glycoprotein M6B in the neurogenic niche and reactive glia in the injury penumbra following traumatic brain injury in mice. Exp. Neurobiol..

[4] Mita S (2015). Transcallosal projections require glycoprotein M6-dependent neurite growth and guidance. Cereb. Cortex.

[5] Fernandez ME, Alfonso J, Brocco MA, Frasch AC (2010). Conserved cellular function and stress-mediated regulation among members of the proteolipid protein family. J. Neurosci. Res..

[6] Drabek K, van de Peppel J, Eijken M, van Leeuwen JP (2011). GPM6B regulates osteoblast function and induction of mineralization by controlling cytoskeleton and matrix vesicle release. J. Bone Miner. Res..

[7] Miao Z (2022). Integrated analysis reveals prognostic value and mesenchymal identity suppression by glycoprotein M6B in glioma. Am. J. Transl. Res..

[8] Nurk S (2022). The complete sequence of a human genome. Science.

[9] Gymrek M (2017). A genomic view of short tandem repeats. Curr. Opin. Genet. Dev..

[10] Ranathunge C (2020). Transcribed microsatellite allele lengths are often correlated with gene expression in natural sunflower populations. Mol. Ecol..

[11] Valipour E (2013). Polymorphic core promoter GA-repeats alter gene expression of the early embryonic developmental genes. Gene.

[12] Arabfard M, Kavousi K, Delbari A, Ohadi M (2018). Link between short tandem repeats and translation initiation site selection. Hum. Genom..

[13] Bushehri A, Barez MR, Mansouri SK, Biglarian A, Ohadi M (2016). Genome-wide identification of human- and primate-specific core promoter short tandem repeats. Gene.

[14] Ohadi M (2015). Core promoter short tandem repeats as evolutionary switch codes for primate speciation. Am. J. Primatol..

[15] Afshar H (2020). Natural selection at the NHLH2 core promoter exceptionally long CA-repeat in human and disease-only genotypes in late-onset neurocognitive disorder. Gerontology.

[16] Mohammadparast S, Bayat H, Biglarian A, Ohadi M (2014). Exceptional expansion and conservation of a CT-repeat complex in the core promoter of PAXBP1 in primates. Am. J. Primatol..

[17] Alizadeh F (2019). Disease-only alleles at the extreme ends of the human ZMYM3 exceptionally long 5' UTR short tandem repeat in bipolar disorder: A pilot study. J. Affect. Disord..

[18] Afshar H (2020). Evolving evidence on a link between the ZMYM3 exceptionally long GA-STR and human cognition. Sci. Rep..

[19] Emamalizadeh B (2017). The human RIT2 core promoter short tandem repeat predominant allele is species-specific in length: A selective advantage for human evolution?. Mol. Genet. Genom..

[20] Jakubosky D (2020). Properties of structural variants and short tandem repeats associated with gene expression and complex traits. Nat. Commun..

[21] Zhang G, Andersen EC (2023). Interplay between polymorphic short tandem repeats and gene expression variation in *Caenorhabditis*
*elegans*. Mol. Biol. Evol..

[22] Horton CA (2023). Short tandem repeats bind transcription factors to tune eukaryotic gene expression. Science.

[23] Lambert SA (2018). The human transcription factors. Cell.

[24] Namdar-Aligoodarzi P (2015). Exceptionally long 5' UTR short tandem repeats specifically linked to primates. Gene.

[25] Ohadi M, Mohammadparast S, Darvish H (2012). Evolutionary trend of exceptionally long human core promoter short tandem repeats. Gene.

[26] Khamse S (2022). Predominant monomorphism of the RIT2 and GPM6B exceptionally long GA blocks in human and enriched divergent alleles in the disease compartment. Genetica.

[27] Bayat H, Omidi M, Rajabibazl M, Sabri S, Rahimpour A (2017). The CRISPR growth spurt: From bench to clinic on versatile small RNAs. J. Microbiol. Biotechnol..

[28] Yameogo P, Gerard C, Majeau N, Tremblay JP (2023). Removal of the GAA repeat in the heart of a Friedreich's ataxia mouse model using CjCas9. Gene Ther..

[29] Lo-Scrudato M (2019). Genome editing of expanded CTG repeats within the human DMPK gene reduces nuclear RNA foci in the muscle of DM1 mice. Mol. Ther..

[30] Shams F (2022). Advance trends in targeting homology-directed repair for accurate gene editing: An inclusive review of small molecules and modified CRISPR-Cas9 systems. Bioimpacts.

[31] Baba Y, Onishi-Sakamoto S, Ide K, Nishifuji K (2022). Nestin is a marker of unipotent embryonic and adult progenitors differentiating into an epithelial cell lineage of the hair follicles. Sci. Rep..

[32] Jurga AM, Paleczna M, Kadluczka J, Kuter KZ (2021). Beyond the GFAP-astrocyte protein markers in the brain. Biomolecules.

[33] Fu JQ (2019). A single factor induces neuronal differentiation to suppress glioma cell growth. CNS Neurosci. Ther..

[34] Thierry-Mieg D, Thierry-Mieg J (2006). AceView: A comprehensive cDNA-supported gene and transcripts annotation. Genome Biol..

[35] Werner HB (2013). A critical role for the cholesterol-associated proteolipids PLP and M6B in myelination of the central nervous system. Glia.

[36] Fjorback AW, Muller HK, Wiborg O (2009). Membrane glycoprotein M6B interacts with the human serotonin transporter. J. Mol. Neurosci..

[37] Sanchez-Roige S (2018). Genome-wide association study of delay discounting in 23,217 adult research participants of European ancestry. Nat. Neurosci..

[38] Wright SE, Todd PK (2023). Native functions of short tandem repeats. Elife.

[39] Marsh S, Hanson B, Wood MJA, Varela MA, Roberts TC (2020). Application of CRISPR-Cas9-mediated genome editing for the treatment of myotonic dystrophy type 1. Mol. Ther..

[40] Batra R (2021). The sustained expression of Cas9 targeting toxic RNAs reverses disease phenotypes in mouse models of myotonic dystrophy type 1. Nat. Biomed. Eng..

[41] Mosbach V (2020). Resection and repair of a Cas9 double-strand break at CTG trinucleotide repeats induces local and extensive chromosomal deletions. PLoS Genet..

[42] Rohrmoser M (2019). MIR sequences recruit zinc finger protein ZNF768 to expressed genes. Nucleic Acids Res..

[43] Leszczynski P (2020). Deletion of the Prdm3 gene causes a neuronal differentiation deficiency in P19 cells. Int. J. Mol. Sci..

[44] Datta TK (2015). Requirement of the transcription factor USF1 in bovine oocyte and early embryonic development. Reproduction.

[45] Di Pietro A (2022). Targeting BMI-1 in B cells restores effective humoral immune responses and controls chronic viral infection. Nat. Immunol..

[46] Li X (2022). Initial activation of STAT2 induced by IAV infection is critical for innate antiviral immunity. Front. Immunol..

[47] Martin M (2011). Cutadapt removes adapter sequences from high-throughput sequencing reads. EMBnet J..

[48] Kim D, Paggi JM, Park C, Bennett C, Salzberg SL (2019). Graph-based genome alignment and genotyping with HISAT2 and HISAT-genotype. Nat. Biotechnol..

[49] Liao Y, Smyth GK, Shi W (2014). featureCounts: An efficient general purpose program for assigning sequence reads to genomic features. Bioinformatics.

[50] Ritchie ME (2015). limma powers differential expression analyses for RNA-sequencing and microarray studies. Nucleic Acids Res..

[51] Leek JT, Johnson WE, Parker HS, Jaffe AE, Storey JD (2012). The sva package for removing batch effects and other unwanted variation in high-throughput experiments. Bioinformatics.

[52] Wickham H (2016). ggplot2: Elegant Graphics for Data Analysis.

[53] Concordet JP, Haeussler M (2018). CRISPOR: Intuitive guide selection for CRISPR/Cas9 genome editing experiments and screens. Nucleic Acids Res..

[54] Bayat H, Modarressi MH, Rahimpour A (2018). The conspicuity of CRISPR-Cpf1 system as a significant breakthrough in genome editing. Curr. Microbiol..

[55] Bayat H, Naderi F, Khan AH, Memarnejadian A, Rahimpour A (2018). The impact of CRISPR-cas system on antiviral therapy. Adv. Pharm. Bull..

[56] Ran FA (2013). Genome engineering using the CRISPR-Cas9 system. Nat. Protoc..

[57] Darbinian N (2021). Cultured cell line models of neuronal differentiation: NT2, PC12, and SK-N-MC. Methods Mol. Biol..

[58] Andrews PW (1984). Retinoic acid induces neuronal differentiation of a cloned human embryonal carcinoma cell line in vitro. Dev. Biol..

[59] Guschin DY (2010). A rapid and general assay for monitoring endogenous gene modification. Methods Mol. Biol..

[60] Bayat H, Pourgholami MH, Rahmani S, Pournajaf S, Mowla SJ (2023). Synthetic miR-21 decoy circularized by tRNA splicing mechanism inhibited tumorigenesis in glioblastoma in vitro and in vivo models. Mol. Ther. Nucleic Acids.

