# Supplementary material for: CRISPR/Cas9-mediated deletion of a GA-repeat in human GPM6B leads to disruption of neural cell differentiation from NT2 cells
Source: Sci Rep. 2024 Jan 25;14:2136. doi: 10.1038/s41598-024-52675-3 (PMC10810867; doi:10.1038/s41598-024-52675-3)
Supplement: Supplementary file 1 — Supplementary Information. [file 41598_2024_52675_MOESM1_ESM.docx]

**Table S1.** RNA-seq datasets

| **Accession** | **Day 0** | **Day 21** |
| --- | --- | --- |
| PRJNA474206 | 0 | 2 |
| GSE229558 | 2 | 0 |
| GSE125370 | 2 | 2 |

**Table S2.** Sequence of all primers and oligonucleotides used in this study.

| **Name** |  | **Sequence** |
| --- | --- | --- |
| GPM6B | F1 | TAAAGCTAGCATAGACAAGGGAGTGGGTTC |
|  | R1 | TAAACAATTGGATTGAAGAAAGGCTGCTC |
| Left gRNA | Top | CACCGAGAATTAATTAGCATTCAT |
|  | Bottom | AAACATGAATGCTAATTAATTCTC |
| Right gRNA | Top | CACCGAAAACAGCCATTCGGCGCC |
|  | Bottom | AAACGGCGCCGAATGGCTGTTTTC |
| GPM6B | F2 | AACACCCCTTCGATTTGGAG |
|  | R2 | TCCAGTTCCTCCTCAGCATTAG |
| GPM6B-real | Forward | AGCAACACTTCTCCACCAAC |
|  | Reverse | TCACCGTGCAGTTCTTTCAC |
| NESTIN | Forward | TGCGGGCTACTGAAAAGTTCC |
|  | Reverse | AGGCTGAGGGACATCTTGAG |
| TUBB3 | Forward | CCCAGTATGAGGGAGATCGTG |
|  | Reverse | GTTCCAGGTCCACCAGAATGG |
| GFAP | Forward | CTGGAGGTTGAGAGGGACAATC |
|  | Reverse | CTCCAGCGACTCAATCTTCCTC |
| MAP2 | Forward | AGGCTGTAGCAGTCCTGAAAGG |
|  | Reverse | CTTCCTCCACTGTGACAGTCTG |
| HPRT | Forward | GCGTCGTGATTAGTGATGATGAA |
|  | Reverse | AGACGTTCAGTCCTGTCCATAA |
| GPM6B-IF | GAAATGAGGTGGGGAGAAAGAG | |
| GPM6B-mutIF | GAAATGAGGTGGGGAGAAATCG | |
| GPM6B-PAM1 | TCATTTCTTCTAGCCAAGTACACC | |
| GPM6B-PAM2 | CATTGAGACTGTAGGCACCAC | |
| GPM6B-mutPAM1 | CACCTCATTTCTTCTAGCCAAGTACTCG | |
| GPM6B-mutPAM2 | GGAGCCGCATTGAGACTGTAGGCACGAG | |
| PX459 Reverse | GCCATTTACCGTAAGTTATGTAACG | |
| eGPM6B | ATTAATTAGCATTCATTGCGTGTACTTGGCTAGAAGAAATGAGGTGGGGAGAAA  TCGAGATTGAGATCTTAAGAAAGAGAGCAAGGAGGAAGAGCCCGGTGGGGAGC  AGGTCCTTCTCCCCCACCCCCCTACCCCCAACTCCCACTGGAGCCGCATTGAGA  CTGTAGGCACCAGCGGGCGCC | |
| eGPM6SB | ATTAATTAGCATTCATTGCGTGTACTTGGCTAGAAGAAATGAGGTGGGGAGAAAG  AGAGAGAGAGAGAGAGATCGAGATTGAGATCTTAAGAAAGAGAGCAAGGAGGAA  GAGCCCGGTGGGGAGCAGGTCCTTCTCCCCCACCCCCCTACCCCCAACTCCCAC  TGGAGCCGCATTGAGACTGTAGGCACCAGCGGGCGCC | |

**Table S3.** Antibodies information

| **Antibody name** | **Immunogen** | **Manufacture, catalog #** | **Raised animal** | **Polyclonal (P)/ Monoclonal (M)** | **Molecular weight** | **Concentration** | **RRID** |
| --- | --- | --- | --- | --- | --- | --- | --- |
| **Western blot- Primary antibodies** | | | | | | | |
| GPM6B | Synthesized peptide derived from human GPM6B | Thermo Fisher Scientific, PA5-68250 | Rabbit | P | 58-62 kDa | 1:1000 | AB_2692045 |
| Nestin (10c2) | Antibody raised against a 150 amino acid epitope mapping near the C-terminus of human nestin | Santa Cruz Biotechnolog, sc-23927 | Mouse | M | 190-200 kDa | 1:1000 | AB_627994 |
| Anti-GFAP | Full length native protein (purified) corresponding to Human GFAP | Abcam, ab7260 | Rabbit | P | 55 kDa | 1:10000 | AB_305808 |
| β3 Tubulin (TU-20) | Antibody raised against amino acids 441-448 of β3 Tubulin of human origin | Santa Cruz Biotechnology, sc-51670 | Mouse | M | 55 kDa | 1:1000 | AB_630408 |
| MAP-2 (A-4) | Antibody raised against amino acids 1-300 of MAP-2 of human origin | Santa Cruz Biotechnology, sc-74421 | Mouse | M | 280 kDa | 1:1000 | AB_1126215 |
| β-Actin (C4) | Antibody raised against gizzard Actin of avian origin | Santa Cruz Biotechnology, sc-47778 | Mouse | M | 43 kDa | 1:1000 | AB_626632 |
| **Western blot- Secondary antibodies** | | | | | | | |
| m-IgGκ BP-HRP | - | Santa Cruz Biotechnology, sc-516102 | Mouse | - | - | 1:10000 | AB_2687626 |
| Anti-rabbit IgG-HRP | - | Santa Cruz Biotechnology, sc-2357 | Mouse | - | - | 1:10000 | AB_628497 |
| **Flowcytometry- Primary antibodies** | | | | | | | |
| Nestin |  | Biorbyt, orb243885 |  |  |  |  |  |
| GFAP |  | Biorbyt, orb401780 | Mouse | M | - | 1:200 | - |
| Beta Tubulin (FITC) | KLH conjugated synthetic peptide derived from human Beta tubulin (61-160/444aa) | Biorbyt, orb464275 | Rabbit | P | - | 1μg/Test | - |
| MAP2 | KLH conjugated synthetic peptide derived from human MAP2 (1-120/1827 aa) | Biorbyt, orb11455 | Rabbit | P | - | 1μg/Test | AB_10751907 |
| **Flowcytometry- Secondary antibodies** | | | | | | | |
| Anti-Mouse IgG(H+L) antibody (FITC) |  | Biorbyt, orb688924 | Goat | P | - | 1:200 | - |
| Anti-Rabbit IgG(H+L) (FITC) |  | Biorbyt, orb688925 | Goat | P | - | 1:200 | - |


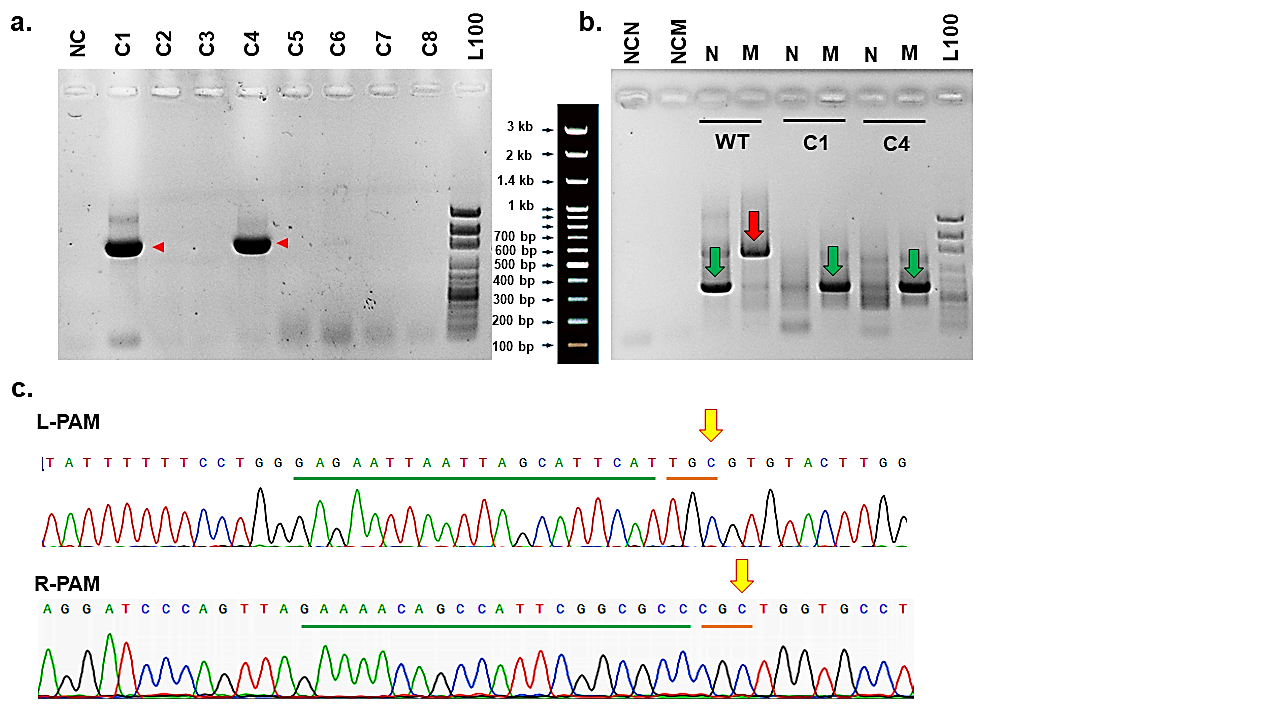


**Figure S1. Using nested ARMS-PCR to select edited clones.** **a**) GPM6B F2 and R2 primer set are located outside of homology arms, to amplify the target site (1543 bp product; indicated with red triangles) and exclude the clones, in which donor template randomly integrated **b**) Nested ARMS-PCR with specific primer sets were used to distinguish edited from unedited clones. In C1 and C4, 670 bp product (indicated with green arrows) was amplified with the primer set that was specific for the deleted allele (M), but were not amplified with the primer set which was specific for the wild-type allele (N). The results of unedited NT2 (WT) cells were vice versa (red arrow is non-specific product). The original gel images are presented in Supplementary Figure Xb and c. **c**) Sanger sequencing confirmed the existence of PAM mutations at gRNA binding sites in edited cells and the scrambled control. Green lines: the location of gRNAs; orange lines: the location of PAM sites; and yellow arrows: the mutated nucleotide at the PAM sites. NC: Negative control; C: Clone; L100: Ladder 100; NCN: Negative control of primer set for wild-type allele; NCM: Negative control of primer set for deleted allele; WT: Wild-type; L-PAM: Left PAM; and R-PAM: Right PAM.

**Supplementary Figures X**

**
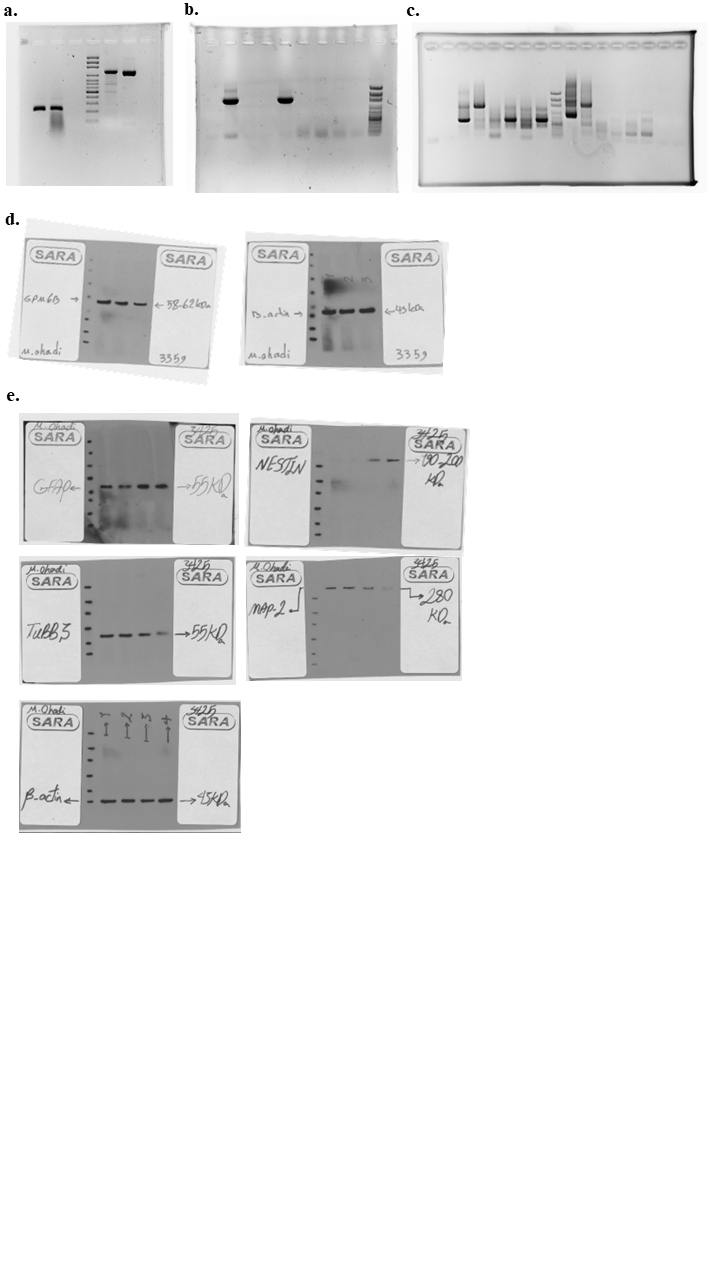
**
